# Depression Severity, Slow- versus Fast-Wave Neural Activity, and Symptoms of Melancholia

**DOI:** 10.3390/brainsci14060607

**Published:** 2024-06-15

**Authors:** Christopher F. Sharpley, Vicki Bitsika, Ian D. Evans, Kirstan A. Vessey, Emmanuel Jesulola, Linda L. Agnew

**Affiliations:** 1 Brain-Behaviour Research Group, University of New England, Armidale, NSW 2351, Australia; vicki.bitsika@une.edu.au (V.B.); ievans3@une.edu.au (I.D.E.); kvessey@une.edu.au (K.A.V.); doctorseasept@yahoo.com (E.J.); linda.agnew@griffith.edu.au (L.L.A.); 2 Department of Neurosurgery, The Alfred Hospital, Melbourne, VIC 3000, Australia; 3 Department of Health, Griffith University, Gold Coast, QLD 4222, Australia

**Keywords:** depression, EEG, melancholia, parietal–occipital

## Abstract

Melancholia is a major and severe subtype of depression, with only limited data regarding its association with neurological phenomena. To extend the current understanding of how particular aspects of melancholia are correlated with brain activity, electroencephalographic data were collected from 100 adults (44 males and 56 females, all aged 18 y or more) and investigated for the association between symptoms of melancholia and the ratios of alpha/beta activity and theta/beta activity at parietal–occipital EEG sites PO1 and PO2. The results indicate differences in these associations according to the depressive status of participants and the particular symptom of melancholia. Depressed participants exhibited meaningfully direct correlations between alpha/beta and theta/beta activity and the feeling that “Others would be better off if I was dead” at PO1, whereas non-depressed participants had significant inverse correlations between theta/beta activity and “Feeling useless and not needed” and “I find it hard to make decisions” at PO1. The results are discussed in terms of the relative levels of fast-wave (beta) versus slow-wave (alpha, theta) activity exhibited by depressed and non-depressed participants in the parietal–occipital region and the cognitive activities that are relevant to that region.

## 1. Introduction

Depression carries a major disease burden [1], and effects about 300 million people worldwide [2]. Effective treatment depends on the accurate diagnosis of depression, but current clinical procedures most commonly rely on clinician interview [3] or patient self-report (e.g., [4,5]), which are open to some degree of subjectivity, with the potential to hinder accurate identification of the presence and severity of depression. Similarly, although it is most commonly referred to as a unitary disorder, Major Depressive Disorder (MDD) is defined by nine Diagnostic Criteria symptoms and many more Associated Features [3], which together can produce 1497 different combinations that qualify for the diagnosis of MDD [6]. As a result of this heterogeneity of MDD, plus the subjectivity of clinical interviews or self-report procedures, much effort has been given to investigating the neurobiological correlates of depression, whatever its symptom profile [7,8]. Different groups of MDD symptoms that qualify for a diagnosis are often referred to as ‘depression subtypes’ [9,10].

One particular subtype of depression that exerts a major burden of suffering and is also resistant to treatment is melancholia [11]. Melancholia is described as including some of the Diagnostic Criteria for MDD as well as additional symptoms [3]. Although there has been some disagreement as to the exact nature of those symptoms [12], Parker and colleagues [13,14,15] have produced a scale that was able to distinguished between melancholic and non-melancholic community participants [15]. Table 1 presents those symptoms, plus items from a standardized scale for measuring depression that match the melancholia symptoms.

The search for neurobiological correlates of melancholia has included studies of the electrical activity of the brain [16], sometimes collected via an electroencephalograph (EEG) [17], which measures electrical activity at sites across the skull, enabling inferences to be drawn regarding brain activity under those sites. However, Bruun et al. [17] found that some methodological limitations in much of the previous research on the neurobiological correlates of melancholia have left this issue yet to be comprehensively resolved.

Using EEG data, there have been some studies of the association between general depression and one commonly used neurobiological index that compares the power of alpha wave activity across the right and left hemispheres of the brain (‘alpha asymmetry’) (reviewed by [18]). Although alpha asymmetry is a valuable research metric, there are other more focused indices of EEG activity that have been applied to studies of MDD and which may be successful in expanding our understanding of how brain activity is associated with melancholia. Some of these metrics are based on the ratio of different frequencies of the brain’s electrical activity. That activity can be classified according to the frequency of the electrical waves measured during consciousness. ‘Theta’ waves in the range of 4 to 8 Hz, ‘alpha’ waves from 8 to 13 Hz, and ‘beta’ waves from 13 to 30 Hz have all been suggested as worthy foci for the study of potential neurobiological markers of depression [19]. Theta waves occur when the individual is quietly focusing upon something, or is lightly asleep; alpha waves occur during relaxation, mostly with eyes closed but not asleep; and beta waves may be observed when the individual is alert or concentrating actively and increase in power during stressful situations, when alpha waves decrease. ‘delta’ waves occur during sleep, ranging from 0.5 to 3.5 Hz, and were not studied here because of the focus upon data collection from awake participants.

These frequency ranges provide an opportunity to compare slow- and fast-wave activity, which can help illuminate brain function and its correlation with other variables such as melancholia. A common method of making those comparisons is to divide the power of the first wave form with that of the second wave form to provide a ratio of the two wave forms. For example, the alpha:beta ratio provides a comparison that is used to study mental attentiveness versus relaxation, so that a low alpha:beta ratio (i.e., greater beta than alpha power) is an indicator of attention [20]. Another such ratio is the theta:beta ratio, which was first thought to indicate a deficit of arousal in attentional disorders, but has since been found to relate more reliably to executive cognitive activity and cognitive processing capacity [21]; a low theta:beta ratio (i.e., greater beta than theta power) has been shown to accompany increased attentional capacity [22].

In a recent EEG study of the neurobiological correlates of melancholia, Sharpley et al. [23] found that only one component of melancholia was inversely associated with alpha asymmetry across several pairs of frontal EEG sites from the right and left hemispheres (F4:F3, FC4:FC3, and FT8:FT7) for depressed participants during eyes-closed resting conditions. Conversely, alpha asymmetry was directly associated with melancholia across two posterior sites (PO2:PO1). PO2 and PO1 are in the parietal–occipital region, which is considered to be involved in the interpretation of visual stimuli [24]. Of relevance to this study, this system was observed in MDD patients to initiate “a counterproductive recruitment of attentional resources” resulting in “dysfunctional network dynamics” ([24], p. 1667). Thus, the importance of the parietal–occipital region, and the PO1 and PO2 sites in particular, is directly relevant to the investigation of slow- versus fast-speed electrical activity and its association with melancholia.

The component of melancholia that was associated with alpha asymmetry in Sharpley et al.’s [23] study was derived via Principal Component Analysis from the eight items depicted in Table 1. It was identified as ‘Difficulties in Social reasoning, Misinterpretation’, and consisted of the four items measuring Anhedonia, Impaired Concentration, and Thoughts of Death (see Table 1). The other component of the eight melancholia items was ‘Fatigue-withdrawal’, and its items were not significantly associated with alpha asymmetry. The results reported by Sharpley et al. [23] for the PO1 and PO2 EEG sites are valuable but do not allow reliable conclusions to be drawn regarding the relative alpha or theta versus beta power in each of these parietal–occipital sites, nor the association between fast- versus slow-wave brain activity and various melancholia symptoms.

Therefore, to extend the findings reported by Sharpley et al. [23], this study analysed data on alpha:beta and theta:beta ratios collected from PO1 and PO2. Using the same research participants for the Sharpley et al. [23] study allowed for a more comprehensive model of the EEG-based neurobiology of melancholia to be developed.

No previous research on the association between alpha:beta and theta:beta ratios at PO1 and PO2 and melancholia was identified (May 2024). However, it is plausible that inverse associations between melancholia and (fast-wave) mental attention and cognitive capacity represent the individual’s intensified brain-related activity aimed at resolving depressive stimuli in a productive manner. Therefore, it was hypothesized that low values in alpha:beta and theta:beta ratios (indicating greater beta power) would be inversely correlated with the severity of melancholia. To further focus this study, only the ‘Difficulties in Social reasoning, Misinterpretation’ items of the melancholia scale were examined, because these were the only items that were previously associated with EEG data (i.e., alpha asymmetry) in Sharpley et al. [23].

## 2. Materials and Methods

### 2.1. Participants

A *priori power analysis* indicated that, to detect a moderate level correlation of at least 0.3, with alpha = 0.05 and power of at least 0.8 [25], 33 depressed participants were needed, which were drawn from a previously described community sample of 100 adults of at least 18 years of age (44 males and 56 females) [23]. These participants were recruited from the New England region of New South Wales following media advertising of the study. Exclusion criteria were as follows: previous medical history of severe physical brain injury, previous brain surgery, or past or current history of epilepsy or seizure disorder. Handedness was not included as a selection criteria because meta-analytic evaluation of data from over 35,000 individuals across 87 studies did not find any evidence of handedness having an effect on depression [26]. Further, although some EEG studies have restricted their samples to right-handed participants, there is no certainty that left hemispheric brain dominance is determined entirely by right handedness, as evidenced by the finding that 61% to 70% of left-handed people also have left hemispheric dominance (Segalowitz and Bryden 1983; Clarke, Howard et al. 2009). There is some evidence that the female menstrual cycle can influence depression [27] and EEG [28], although there is also evidence to the contrary [29] and some data which suggest that this effect is most often observed in the EEG alpha wave signal from frontal regions of the brain [30] (which were not collected in this study), or is part of a relationship between the menstrual cycle and related mood disorders [31]. Significant proportions of the current sample of females refused a question regarding their menstrual cycle or their contraceptive habits and also refused a salivary test for luteal phase. Therefore, this information was not able to be collected.

### 2.2. Instruments

#### 2.2.1. Depression: Melancholia

In addition to a background questionnaire (age sex), participants completed the 20-item Self-rating Depression Scale (SDS), plus one extra item “I do not feel much better even when good things happen”, based on Parker [15]. These items were responded to by participants according to the standard SDS criteria (‘None or a little of the time’ = 1, ‘Some of the time’ = 2, ‘Good part of the time’ = 3, or ‘Most or all of the time’ = 4). The 20 SDSs give a total raw score of between 20 and 80, with a cutoff of 40 or above to denote “clinically significant depression” ([32], p. 335). The SDS has demonstrated split-half reliability of 0.81 [4], 0.79 [33], and 0.94 [34] and internal consistency (alpha) of 0.88 for depressed patients and 0.93 for non-depressed patients [35]. Participants were classified into depressed’ or ‘non-depressed’ on the basis of Zung’s cutoff score of at least 40. From these twenty SDS items, seven items plus the extra item based on Parker [15] were used to form a Melancholia scale (MEL), as shown in Table 1. In their previous report on these data, Sharpley et al. [23] found that these eight items fell into two discrete factors when subject to Principal Component Analysis, so that the first four items shown in Table 1 were identified as ‘Fatigue-Withdrawal” (Factor 1) and the last 4 items in Table 1 were named ‘Difficulties in Social Reasoning, Misinterpretation’ (Factor 2).

#### 2.2.2. EEG Data

EEG data were collected using a 40-channel Digital EEG Amplifier (NuAmps) and a *Quick Cap* with electrodes, during the 3 min eyes closed resting condition. Participants’ hair had been washed with a normal shampoo before the EEG session, and they had all refrained from caffeine or other stimulants for at least 12 h before data collection. Electrode sites were cleaned with *Nuprep* gel before cap fitting, with the Cz electrode located half-way between the glabella and the inion. Data were collected with the *Curry 7* software while participants sat in a soundproofed booth.

Based on Oostenveld and Praamstra’s [36] extended 10–20 placement system, 24 active homologous EEG channels were used in the Sharpley et al. [23], but data from only the two parietal–occipital lobe electrodes PO1 (left hemisphere) and PO2 (right hemisphere) were included in this study. Referencing electrodes were the ground electrode (GND), the central electrodes (Fz, FCz, Cz, CPz, and Oz), ear (Auricle) electrodes (A1 and A2), the Horizontal Electro-Occulographic electrodes (X2 and X4), and the Vertical Electro-Occulographic electrodes (X1 and X3).

Data were collected at theta, alpha, and beta frequency ranges via online filters, at a sampling rate of 1 KHz, with all electrodes having a maximum impedance of 5 kΩ. Theta was defined as having a frequency of 4.0 to 7.9 Hz, ‘alpha’ waves from 8.0 to 12.9 Hz, and ‘beta’ waves from 13.0 to 30.0 Hz. The CAR (Common Average Referencing) style was applied, and EEG data were processed with a band pass low-filter (high-pass) frequency of 1 Hz and a slope of 2 Hz, and a high-filter (low-pass) frequency of 30 Hz and a slope of 8 Hz. A notch filter of 50 Hz (harmonics) with a slope of 1.5 Hz was also used, plus a band stop filter frequency of 50 Hz (harmonics) with a width of 10 Hz and slope of 5 Hz. A Hann window was used to taper the data, using a 10% width to prevent data loss. After collection, EEG data were visually examined and any artefacts (eye movements, muscle movements, spontaneous discharges, or electrode pops, etc), were removed. Further artefact detection was carried out by automatic bad block detection and eye blink detection (using the magnitude of eye blink deflections as a set threshold criteria to detect artefacts). Artefact reduction was conducted by three automated methods (i.e., subtraction, covariance, and Principal Component Analysis) to produce clean EEG data. Back-to-back epochs of 4 s duration were created from the cleaned EEG data, and spectral analysis with a Fast Fourier Transformation was used to calculate the power spectra across the 4 s epochs. From these data, the total power within each of the theta, alpha, and beta ranges was calculated for each participant and transferred to SPSS 27 for statistical analysis. Ratio data were calculated by dividing the first wave frequency by the second wave frequency for each ratio pair (i.e., alpha:beta and theta:beta).

### 2.3. Procedure

After reading an Explanatory Statement and completing a Consent Form, participants completed the background questionnaire, SDS, MEL, and had their scalps prepared and the EEG cap and electrodes fitted before entering the experimental booth. They were fitted with headphones to deliver instructions and minimize external noise. Participants were asked to relax. They then experienced a 15 min adaptation period, followed by an audio-recorded experimental protocol for 3 min of the eyes closed condition. After the protocol, participants departed from the experimental booth and had the electrodes and cap removed. Ethics approval for this protocol was received from the University of New England Human Research Ethics Committee (Approval No. HE14–051).

### 2.4. Statistical Analyses

Scale internal consistency and data normality were calculated. Differences between males and females and depressed and non-depressed participants for their SDS and MEL scale scores and EEG data were tested by MANOVA, using the Type II Sums of Squares and Pillai’s Trace where subsample sizes were unequal [37]. Because of nonnormality in the EEG data, Spearman’s correlation coefficients were used to detect associations between the EEG ratio data and the SDS and MEL scores and MANOVA was used to detect differences between the depressed and non-depressed participants and between males and females, because MANOVA is also robust to the effects of non-normality [37]. These correlation coefficients were calculated for the non-depressed participants and for the depressed participants. Because of the number of correlation coefficients calculated, and the risk of a Type I error, the indicator of a meaningful result was based upon (i) the traditional level of significance of *p* < 0.05, plus (ii) the presence of a correlation of at least 0.3 (i.e., a ‘medium’ strength association [25]) rather than a simple correction to *p* values (e.g., Bonferroni), which may have increased the likelihood of a Type II error [38,39].

## 3. Results

### 3.1. Data

The internal consistency (Cronbach alpha) was 0.921 for the SDS and 0.877 for the eight-item MEL scale. There were 33 participants who had SDS scores in the clinically significant range (referred to as ‘depressed’), and 67 who were able to be classified as non-depressed, using Zung’s [32] cutoff raw score of 40. There were no significant differences between the males’ and females’ SDS or MEL scores (both *p* > 0.6), or between their EEG data for either of the PO1 or PO2 sites (all *p* > 0.251). As mentioned above (Section 2.1), luteal phase data were not able to be ascertained from the females, and so their age was recategorized into subgroups of 18–30 y, 31–40 y, 41–50 y, and 51+ y as a less direct indicator of the presence of a menstrual cycle according to observed age range changes for this physiological variable [40]. The MANOVA indicated no significant differences between the SDS, MEL, or any of the EEG data between these four subgroups of females, suggesting that the presence of a menstrual cycle (and, therefore, luteal phase) did not significantly influence the major dependent variables. Depressed participants had significantly higher SDS (*F*(1,99) = 273.729, *p* < 0.001, η_p_^2^ = 0.736), total MEL (*F* = 208.366, *p* < 0.001, η_p_^2^ = 0.680), and individual MEL item scores (all *F* > 29.00, *p* < 0.001, η_p_^2^ > 0.55) than the non-depressed participants. There was no significant correlation between participants’ ages and their SDS (*r* = 0.055) or MEL (*r* = 0.097) scores.

### 3.2. EEG Ratio Data and MEL Scores

**Figure 1:**
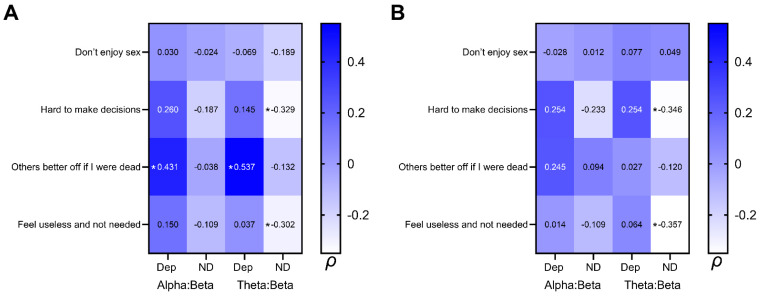
*Heat maps* of Spearman correlation coefficients (ρ) between MEL items and alpha:beta and theta:beta ratios at PO1 (**A**) and PO2 (**B**). * Met criteria for meaningful association.

There were no meaningful correlations between the MEL items and any of the EEG ratios. As mentioned above, Sharpley et al. [23] reported that only MEL Factor 2 (‘Difficulties in Social reasoning, Misinterpretation’, consisting of the four items measuring Anhedonia, Impaired Concentration, and Thoughts of Death; see Table 1) showed any significant association with alpha asymmetry. Table 2 presents the Spearman correlations between the alpha:beta and theta:beta ratios and these four MEL items. Meaningful coefficients are shown in bold and underlined. Although only six of the possible coefficients were meaningful per the criteria set here (i.e., a coefficient of at least 0.3, plus *p* < 0.05), Table 2 presents all the correlation coefficients between the four MEL items and the multiple indices of EEG activity in order to provide a comprehensive account of the complete set of associations between these aspects of Melancholia and slow/fast wave activity. Figure 1 presents these associations in heat map graphic format for easier understanding of the associations between these EEG and MEL variables.

When comparing the meaningful associations between these MEL Factor 2 items and PO1 EEG ratios for the depressed versus non-depressed participants, the major difference is in the direction of the correlation coefficient. That is, for the depressed participants, these correlation coefficients were positive (Figure 1A, *Others better off if I were dead*: 0.431 alpha:beta and 0.537 theta:beta). In contrast, for the non-depressed participants there were only meaningful negative correlations, and these were observed for PO1 and PO2 (Figure 1A and Figure 1B, respectively, *Hard to make decisions* and *Feel useless and not needed*). Thus, not only were the correlations between MEL items in a different direction for the depressed versus non-depressed participants, but the specific aspects of Melancholia that were meaningfully associated with these EEG ratios were also different.

Although not of critical value, it is also noteworthy that 14 of the 16 (87.5%) coefficients for the depressed participants were also direct, and 13 of the 16 (81.3%) coefficients for the non-depressed participants were also inverse, suggestive of a degree of consistency across all the associations between these MEL items and the EEG ratio data for the parietal–occipital lobes, regardless of their statistical power.

None of the PO2 site EEG ratio data were meaningfully associated with MEL items for the depressed participants. This isolation of the EEG ratio:MEL item association to PO1 for the depressed participants argues for a very specific brain region activity association with Melancholia symptoms for the depressed participants that was present at a meaningful level in the left hemisphere only, whereas the non-depressed participants showed a meaningful EEG ratio/MEL item association that was recorded across both hemispheres.

**Figure 2:**
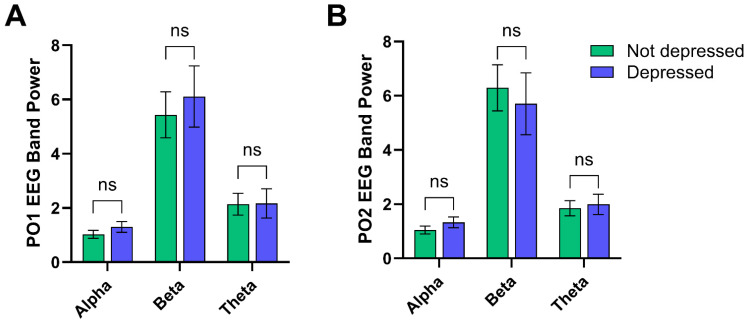
Mean (SE) for alpha, beta, and theta power (10×log10µV^2^) for PO1 (**A**) and PO2 (**B**) for depressed and non-depressed participants.

Finally, it was suggested by Parker et al. [13,15] that overall depression severity can affect the severity of Melancholia. The presence of meaningful correlations between EEG ratios and MEL items, therefore, may have been due to (a) the differences in those item scores between depressed and non-depressed participants reported above, (b) the EEG ratios alone with no reference to MEL severity, or (c) the influence of both of these. Comparison of the alpha, beta, and theta wave power at the PO1 and PO2 sites for depressed versus non-depressed participants via MANOVA revealed a nonsignificant main effect (*F*(6,77) = 1.411, *p* = 0.221, η_p_^2^ = 0.099), and no significant univariate effects (all *p* > 0.253, η_p_^2^ < 0.016) (see Figure 2). These findings support Parker et al.’s [13,15] argument that the severity of depression is a major influence upon Melancholia.

## 4. Discussion

The results of this investigation of slow- versus fast-wave activity in the parietal–occipital region and its association with one aspect of Melancholia provide further information about the way that specific symptoms of depression are associated with brain activity in the parietal–occipital region. Four major findings emerged from this study. First, depressed and non-depressed participants’ associations with some symptoms of Melancholia were in different directions; second, different aspects of slow/fast wave activity were associated with discrete symptoms of melancholia; third, depressive status dictated whether the associations between the EEG ratios and symptoms of Melancholia were noted at one, or two, parietal–occipital sites; and fourth, these results were governed by the depressive state of the participants. These findings argue for an interaction between depressive state, EEG sites, and melancholia symptoms, thus underling the complexity of the brain activity and Melancholia relationship.

Because PO2 associations between the theta:beta ratios and Melancholia symptoms were confined to the depressed participants, PO1 represents the most informative region for distinguishing between depressed and non-depressed participants. It is, therefore, relevant to consider the possible functions of the parietal–occipital region that may be relevant to melancholia. The parietal–occipital region has been found to be active during tasks concerned with paying visual attention to stimuli, and is implicated in deficient attentional filtering of information that may occur in depressed patients [24]. An individual’s visual memory working capacity can predict several cognitive functions, mostly via the intraparietal sulcus and its ability to influence personal representations of reality [41]. In depressed patients, this region may be responsible for them misinterpreting their interpersonal environment so that they might believe that *Others would be better off if I were dead*, or that they were seen by others as *useless* and, therefore, *not needed*.

The finding that the depressed participants in this study exhibited meaningful direct correlations between their Thoughts of Death or Suicide (*Others would be better off if I were dead*) and their alpha:beta ratio and their theta:beta ratio at PO1 suggests that these depressed participants showed more slow-wave and less fast-wave activity in the region where they were likely to pay attention to their visual interpersonal environment. By contrast, the non-depressed participants showed an inverse correlation between their theta:beta ratio, suggestive of more fast-wave activity underlying their ability to make decisions about that visual environment and their feeling of being useless and not needed. Because fast-wave activity (i.e., beta wave activity in this study) is generally associated with being alert or concentrating actively, it is plausible that depressed persons may exert less focused concentration and critical problem-solving activity when confronted with the possible thoughts of being unwanted by others, instead lapsing into brain wave activity more usually occurring under conditions of near-sleep. Although hypothetical at this stage, this explanation of the current findings fits with the underlying cognitive processes of feeling socially misunderstood [42] or experiencing a mismatch between the world as is experienced by depressed people [43]. Other hypotheses that might be entertained would rely upon the construction of experimental conditions that require participants to perform a cognitive task, such as the odd-ball challenge [44]. This kind of extension to the current study could help develop a more comprehensive model of the associations noted herein.

Given that the parietal–occipital region has a plausible role in depressive cognitions, and that alpha power is reduced bilaterally when encoding visual information [45,46], it is of interest that the meaningful associations for the depressed participants were confined to only one hemisphere in that region (i.e., the left hemisphere, PO1), whereas non-depressed participants’ associations with MEL symptoms were linked with both hemispheres (i.e., PO1 and PO2, and in the same direction that was indicative of higher beta power than alpha or theta power). However, depressed participants also showed lower beta activity and higher alpha and theta activity at their PO1 site, suggestive of a lower intensity of (beta-wave) cognitive activity at PO1. Although it did not reach the level of meaningfulness required in this study, depressed participants’ beta activity at PO2 was also relatively low when associated with the MEL item regarding feelings of worthlessness. Thus, the entire parietal–occipital region might be characterized by lower beta activity for depressed participants, although more powerfully in PO1. This region is involved in focusing upon important environmental stimuli *vis à vis* the internal goals of the individual [47] and then using that sensory information to make decisions [48]. The parietal–occipital region is also involved in the coding of episodic memory [49], potentially confirming the negative emotional response to others’ evaluations of one’s self, and contributing to the poor self-evaluation that may result in feeling *useless and not needed*. These points reiterate the complexity of the role the parietal–occipital region plays in evaluating external stimuli, which may include perceptions of how others evaluate an individual, thus relating to misinterpretations of those evaluations, resulting in belief that *Others would be better off if I were dead.*

Although these results are initial, and require confirmation and further exploration, they are consistent with a recent report that direct current stimulation to the dorsolateral prefrontal cortex produced increases in spectral power between 6 Hz and 30 Hz in the parietal–occipital region, accompanied by significant increases in clinical indices of improvement in a sample of Disorders of Consciousness patients [50]. Although no depression data were reported in that study, the link between increases in parietal–occipital brain activity and a reduction in Disorder of Consciousness symptoms provides some support for the parietal–occipital region’s role in a range of behaviours congruent with the ability to understand the external environment, including others’ perceptions of one’s self that may contribute to lower estimations of the value of that self to society.

Several limitations of this research must be mentioned. The sample was of satisfactory size for data analysis, but replication with larger samples would add statistical power and potentially uncover further details regarding the links between PO1 and PO2 brain activity and aspects of Melancholia. Although explained in the Introduction as an extension of a previous study on Melancholia [23], and also because of the relevance of the parietal–occipital lobe to depression [24], the restriction of EEG sites investigated to just PO1 and PO2 is a limitation, and extension of this study to include other areas of the brain may be of value. The choice of alpha, beta, and theta waves was argued in the Introduction, but the inclusion of other wave bands, or the division of alpha and beta into sub-bands, may also help provide a more comprehensive model of brain electrical activity, although several studies have failed to find any significant difference in the associations between alpha wave band activity when it was measured via the 8–12.99 Hz method or via individual peak alpha frequency [51,52]. Female luteal phase data were not able to be collected, but analysis of depression, Melancholia, and EEG data across age subgroups of females did not indicate the presence of any significant differences that might have occurred if aspects of the intact menstrual cycle were acting upon these variables. Although not conclusive, these results do not reflect a significant confound from the presence/absence of the female menstrual cycle (or any parts of that cycle). Although the lack of luteal phase data from those females in this study who were cycling is a limitation, recent findings report a lack of any significant effects of the luteal phase upon alpha power or peak frequency [29]. Participation was voluntary, and no attempt was made to recruit participants who were suffering from very restrictive depression; the inclusion of such a comparative subsample would further test the validity of these initial findings. The measure of depression and MEL were self-report, and confirmation with clinical interviews would boost the validity of these data. The study was a snap-shot, and did not collect data over a period of time, which could contribute to understanding how depressed persons might vary in their association between specific aspects of melancholia and the kinds of cognitive functions based in the parietal–occipital region. The differences in MEL items that were associated with PO1 fast–slow wave activity by the depressed versus non-depressed participants needs to be better understood before a complete picture of this phenomenon can be drawn.

## 5. Conclusions

Notwithstanding these limitations, the results of this study provide some further understanding of how aspects of Melancholia are represented in fast- versus slow-wave brain activity. The overall finding (based upon the direction of the correlation coefficients depicted in Table 2) was that depressed participants exhibited less fast-wave activity than non-depressed participants. This finding is relevant to therapy settings when working with depressed patients who exhibit depression and Melancholia, and who report that they have feelings of uselessness, being under-valued by others, and have an inability to make effective decisions. These patients may benefit from training to increase their (beta-related) discriminatory cognitive activity to refute those mistaken beliefs.

## Figures and Tables

**Table 1:** Melancholia symptoms and items ^1^.

Melancholia Symptoms	Low Energy	Loss of Interest	Impaired Concentration	Lack of Improvement in Mood	Anhedonia	Impaired Concentration	Thoughts of Death/Suicide	Thoughts of Death/Suicide
Items ^2^	Feel tired for no reason	Do not enjoy doing the things I used to	Mind is unclear	Do not feel better when good things happen	Do not enjoy sex	Hard to make decision	Others better off if I were dead	Feel useless and not needed

^1^ Based on Parker et al. [13,15]. ^2^ Derived from the Self-rating Depression Scale [4].

**Table 2:** Meaningful ^1^ (bold, underlined) Spearman correlations between MEL ^2^ factor 2 items ^3^ and alpha:beta and theta:beta ratios at selected EEG sites for 33 depressed and 67 non-depressed participants.

Melancholia Symptoms and Items	Anhedonia *Don’t Enjoy Sex*				Impaired Concentration *Hard to Make Decisions*				Thoughts of Death/Suicide *Others Better Off If I Were Dead*				Thoughts of Death/Suicide *Feel Useless and Not Needed*			
EEG sites	Alpha:Beta		Theta:Beta		Alpha:Beta		Theta:Beta		Alpha:Beta		Theta:Beta		Alpha:Beta		Theta:Beta	
	Dep ^4^	ND ^5^	Dep	ND	Dep	ND	Dep	ND	Dep	ND	Dep	ND	Dep	ND	Dep	ND
PO1 PO2	0.030 −0.028	−0.024 0.012	−0.069 0.077	−0.189 0.049	0.260 0.254	−0.187 −0.233	0.145 0.254	**−0.329** **−0.346**	**0.431** 0.245	−0.038 0.094	**0.537** 0.027	−0.132 −0.120	0.150 0.014	−0.109 −0.109	0.037 0.064	**−0.302** **−0.357**

^1^ *ρ* ≥ 0.3 and *p* < 0.05. ^2^ MEL Factor 2: Difficulties in Social Reasoning. ^3^ Based on Parker et al. [13,15] and derived from the SDS [4], ^4^ D = Depressed. ^5^ ND = Not Depressed.

## Data Availability

The original contributions presented in this study are included in the article; further inquiries can be directed to the corresponding author.
